# Over 1000 nm Near-Infrared Multispectral Imaging System for Laparoscopic In Vivo Imaging

**DOI:** 10.3390/s21082649

**Published:** 2021-04-09

**Authors:** Toshihiro Takamatsu, Yuichi Kitagawa, Kohei Akimoto, Ren Iwanami, Yuto Endo, Kenji Takashima, Kyohei Okubo, Masakazu Umezawa, Takeshi Kuwata, Daiki Sato, Tomohiro Kadota, Tomohiro Mitsui, Hiroaki Ikematsu, Hideo Yokota, Kohei Soga, Hiroshi Takemura

**Affiliations:** 1 Exploratory Oncology Research & Clinical Trial Center, National Cancer Center, Kashiwa, Chiba 277-8577, Japan; hikemats@east.ncc.go.jp; 2 Research Institute for Biomedical Sciences, Tokyo University of Science, Noda, Chiba 278-8510, Japan; mail@ksoga.com (K.S.); takemura@rs.tus.ac.jp (H.T.); 3 Department of Materials Science and Technology, Tokyo University of Science, Katsushika, Tokyo 162-8601, Japan; 8219518@ed.tus.ac.jp (Y.K.); 8220505@ed.tus.ac.jp (R.I.); kyohei.okubo@rs.tus.ac.jp (K.O.); masa-ume@rs.tus.ac.jp (M.U.); 4 Department of Mechanical Engineering, Tokyo University of Science, Noda, Chiba 278-8510, Japan; 7519502@ed.tus.ac.jp (K.A.); 7520507@ed.tus.ac.jp (Y.E.); 5 Department of Gastroenterology and Endoscopy, National Cancer Center Hospital East, Kashiwa, Chiba 277-8577, Japan; ktakashi@east.ncc.go.jp (K.T.); dsatou@east.ncc.go.jp (D.S.); tkadota@east.ncc.go.jp (T.K.); tmitsui@east.ncc.go.jp (T.M.); 6 Department of Pathology and Clinical Laboratories, National Cancer Center Hospital East, Kashiwa, Chiba 277-8577, Japan; tkuwata@east.ncc.go.jp; 7 RIKEN Center for Advanced Photonics, Wako, Saitama 351-0198, Japan; hyokota@riken.jp

**Keywords:** laparoscope, InGaAs camera, multispectral imaging, near infrared, live imaging

## Abstract

In this study, a laparoscopic imaging device and a light source able to select wavelengths by bandpass filters were developed to perform multispectral imaging (MSI) using over 1000 nm near-infrared (OTN-NIR) on regions under a laparoscope. Subsequently, MSI (wavelengths: 1000–1400 nm) was performed using the built device on nine live mice before and after tumor implantation. The normal and tumor pixels captured within the mice were used as teaching data sets, and the tumor-implanted mice data were classified using a neural network applied following a leave-one-out cross-validation procedure. The system provided a specificity of 89.5%, a sensitivity of 53.5%, and an accuracy of 87.8% for subcutaneous tumor discrimination. Aggregated true-positive (TP) pixels were confirmed in all tumor-implanted mice, which indicated that the laparoscopic OTN-NIR MSI could potentially be applied in vivo for classifying target lesions such as cancer in deep tissues.

## 1. Introduction

The number of cancer cases and deaths worldwide has been strikingly increasing with the rapidly aging population [1]. Surgical resection is one of the most common procedures used to treat cancers. As a minimally invasive surgery compared to laparotomy, laparoscopic surgery has been in great demand because the need for such surgeries has increased. Therefore, in order to perform laparoscopic surgery more safely and efficiently, it is necessary to develop an image-guided surgical support system identifying cancer-associated regions and anatomical structures such as blood vessels and nerves.

Recent advances in imaging technologies associated with machine learning have led to the identification of anatomical structures and the localization of cancer in surgical image data [2,3]. However, red, green, and blue (RGB) images acquired by standard visible-light-sensitive cameras only allow the observation of the target’s surface. In addition, it is challenging to distinguish between tissues with similar colors and shapes. Therefore, the use of spectral information beyond the visible light wavelength range in the learning process plays a crucial role in machine learning approaches to catch up with, or even outperform, predictions made by medical experts.

Near-infrared (NIR) light offers advantages over the visible spectral range, including high biological transparency owing to its low absorption and scattering by tissues. The over 1000 nm (OTN) wavelength NIR region (1000–2000 nm), often called the “second biological window” [4], provides higher penetration depth down to 10 mm [5], as well as chemical information corresponding to the overtone and combination tone of molecular vibrations. This slight absorption, which is at a magnitude 10^−2^ lower than that of the visible or mid-infrared regions [6], delivers the fingerprint of chemical composition in the deep tissue [7]. Moreover, spectral imaging, which can acquire the spectrum of each camera pixel, can analyze a large amount of data with a high spatial resolution [8]. Spectral imaging with a high wavelength resolution is called hyperspectral imaging (HSI) [9,10]. Various resected cancers have been analyzed using HSI [11,12,13,14,15,16]. For instance, Sato et al. reported that NIR-HSI could be used to identify tumors covered by normal mucosa [17]. To apply NIR-HSI to real-time imaging, wavelength bands must be reduced because the HSI data (200–300 bands) require substantial processing time. Therefore, Akimoto et al. proposed a selection method for valuable wavelengths, and the authors indicated that four bands in the range of 1000–1400 nm could identify the region of a gastrointestinal stromal tumor under normal mucosa, using the same level of accuracy as NIR-HSI [18]. The results indicate that multispectral imaging (MSI) using selected wavelengths in the range of OTN-NIR could potentially recognize invisible cancer and organs during surgery without labeling with a fluorescent probe. However, to date, only studies on laparoscopic HSI or MSI using less than 1000 nm light have been reported [19,20,21].

In this study, based on a laparoscope that can observe OTN-NIR images, as reported by Zako et al. [22], a wavelength-selectable light source and an optical system for laparoscopy were built to perform OTN-NIR MSI with easy operability. We demonstrate the proof-of-concept for this prototype laparoscope and light source.

## 2. Materials and Methods

### 2.1. Light Source and Laparoscope for OTN-NIR Multispectral Imaging

**Figure 1:**
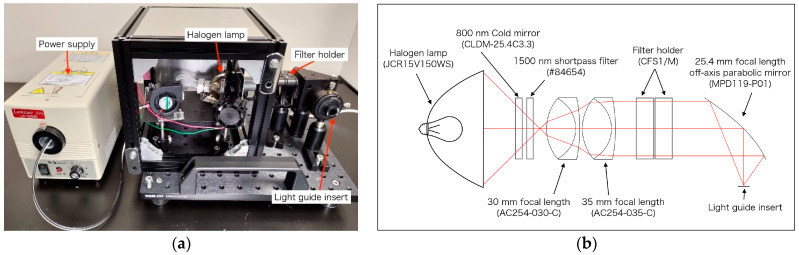
(**a**) Developed light source for over 1000 nm (OTN)-near-infrared (NIR) multispectral imaging; (**b**) optical ray tracing at 1200 nm for the light source optical system.

In this study, a light source that can perform OTN-NIR multispectral imaging was developed. In Figure 1a, the developed light source able to select the output wavelength manually using an NIR bandpass filter is shown. The light source was constructed with a cold mirror (CLDM-25.4C3.3, SIGMA KOKI, Tokyo, Japan), a 1500 nm shortpass filter (#84654, Edmund Optics, Barrington, NJ, USA), two achromatic lenses (AC254-030-C and AC254-035-C, Thorlabs, Newton, NJ, USA), and an off-axis parabolic mirror (MPD119-P01, Thorlabs, USA) based on a 150 W halogen lamp (JCR15V150WS and LA-150UE, HAYASHI-REPIC CO., LTD., Tokyo, Japan). The filter holders (CFS1/M, Thorlabs, USA), inserted in the light source, enabled the selection of the output wavelength. The optical system, modeled using the CODEV optical ray-tracing software, is shown in Figure 1b. As the selectable filters, 14 wavelength (1000–1400 nm) filters that were commercially available are listed in Table 1. For filters below 1150 nm, a 1250 nm shortpass filter was added because transmitted light was observed in the region over 1400 nm.

**Figure 2:**
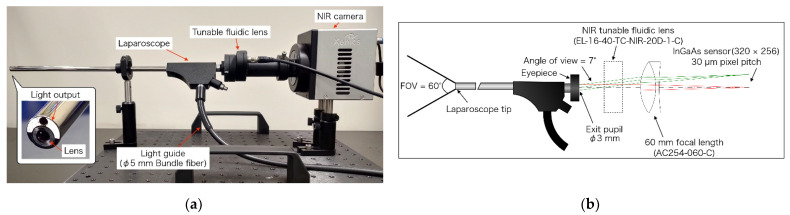
(**a**) Laparoscopic OTN-NIR imaging device; (**b**) optical ray tracing at 1300 nm for a laparoscopic optical system. The minimum (0°) and maximum (7°) angles of view are shown in red and green. The FOV was 60°.

The prototype imaging system developed to perform OTN-NIR multispectral imaging under a laparoscope is depicted in Figure 2a. The device was constructed with a custom-made VIS-NIR endoscope (Machida Endoscope Co., Tokyo, Japan) [22], an NIR tunable fluidic lens (EL-16-40-TC-NIR-20D-1-C, Optotune, Dietikon, Switzerland), an achromatic lens (AC254-060-C, Thorlabs, Newton, NJ, USA), and a 320 × 256 pixel InGaAs camera (Xeva-1.7-320, Xenics, Leuven, Belgium). The optical system is illustrated in Figure 2b. As shown in the figure, the field of view (FOV) was approximately 60°, the exit pupil diameter was 3 mm, and the maximum eyepiece field angle was ±7°. The laparoscope had a light guide of 5 mm diameter bundle fiber connecting to the light source. Due to a focus shift that occurred when the wavelength was changed in the light source, the optical power of the tunable lens was set, as shown in Table 1, to adjust the focus [23]. By tuning the optical power, the angle of view changed on the order of several pixels. Therefore, based on the image at 1000 nm, which had the largest angle of view, all wavelength images were enlarged, and the positions were matched by an affine transformation.

### 2.2. Properties of the Laparoscope and Light Source

**Figure 3:**
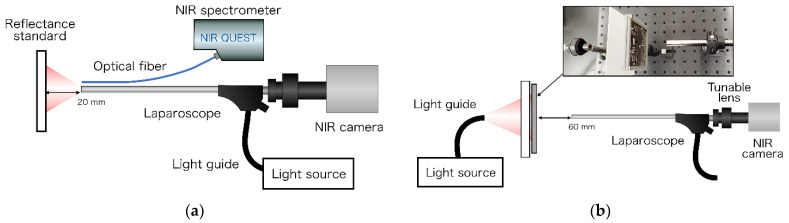
(**a**) Spectroscopy for the light source of the laparoscopic OTN-NIR multispectral imaging system. The exposure time was set to 500 ms. The average was 10 times. (**b**) Resolution evaluation of the laparoscopic NIR imaging device.

The spectral characteristics of the developed light source at 14 wavelengths were confirmed using a 99% reflectance standard (SG3052, SphereOptics GmbH, Herrsching, Germany) placed at 20 mm from the tip of the laparoscope. Subsequently, the spectrum was measured using an NIR spectrometer (NIRQuest512-2.2, Ocean Photonics, Tokyo, Japan), as shown in Figure 3a.

The resolution using the developed laparoscopic OTN-NIR imaging device was evaluated using a 1951 USAF resolution chart (R3L3S1N, Thorlabs) placed at 60 mm from the laparoscope tip. Illumination was provided from outside to ensure even lighting. This light transilluminated a 100 × 100 mm N-BK7 ground glass diffuser (DG100X100-220, Thorlabs, Newton, NJ, USA) onto which the resolution chart was placed. The contrast for each element of the USAF resolution chart was calculated using the Michelson contrast formula, as follows [24]:

(1) $$Contrast\;=\;\frac{I_{max}-I_{min}}{I_{max}+I_{min}}$$

where $I_{max}$ and $I_{min}$ represent the highest and lowest pixel intensity values of the adjacent bars within an element, respectively.

### 2.3. OTN-NIR Multispectral Imaging for Live Mouse

**Figure 4:**
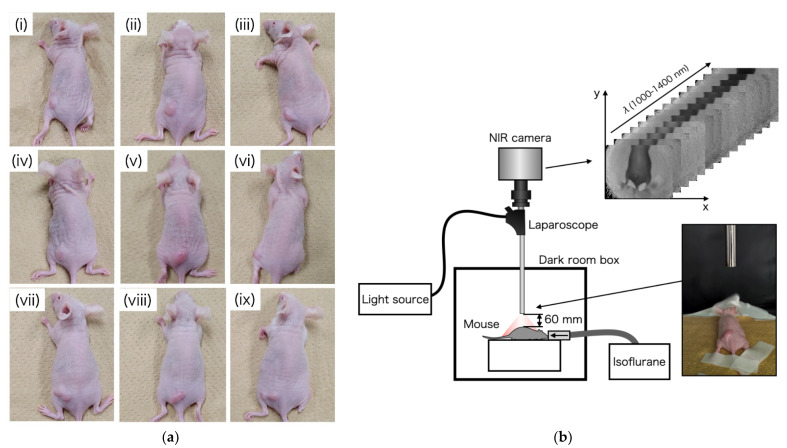
(**a**) Tumor-bearing mice on day 14, after implantation of HT29 cells; (**b**) experimental setup of OTN-NIR multispectral imaging under laparoscope.

To prepare tumor-bearing mice, HT29 human colon cancer cells (ATCC HTB-38) were cultured in Roswell Park Memorial Institute medium (RPMI 189-02025, FUJIFILM Wako Pure Chemical Corporation, Osaka, Japan) supplemented with 10% fetal bovine serum (FBS 10270-106, Gibco) and 1% penicillin/streptomycin (161-23181, FUJIFILM Wako Pure Chemical Corporation). All cells were maintained at 37 °C and 5% CO_2_ in a humidified incubator. HT29 cells (1 × 10^7^) were implanted in the left waist of nine female BALB/c-nu nude mice at 6 weeks of age (Charles River Laboratories Japan, Inc., Kanagawa, Japan). Mice 14 days after cancer implantation are shown in Figure 4a.

In this study, OTN-NIR multispectral imaging under a laparoscope was performed in a dark box, as shown in Figure 4b. The OTN-NIR multispectral images were captured for the live mice before tumor implantation and 14 days post-implantation. The distance between the tip of the laparoscope and the mouse was 60 mm. All procedures and protocols were approved by the Animal Care and Use Committee of the National Cancer Center (K19-016).

### 2.4. Data Processing

It was necessary to calibrate images from the dark noise and white standard in each pixel (i,j) to analyze each mouse image. Moreover, in this study, each reflectance was converted to absorbance. The calibration was performed as follows:

(2) $$A\left(i,j\right)=-\log_{10}\left(\frac{I_{r}\left(i,j\right)-I_{d}\left(i,j\right)}{I_{w}\left(i,j\right)-I_{d}\left(i,j\right)}\right)$$

where A(i,j) is the row vector of the absorbance spectrum for the obtained image, and $I_{r}\left(i,j\right)$, $I_{w}\left(i,j\right)$, and $I_{d}\left(i,j\right)$ are row vectors of raw data, white standard data, and dark data, respectively. When $I_{w}\left(i,j\right)-I_{d}\left(i,j\right)$ was lower than zero, the pixel was excluded from the calculation. Because the pixel data had a 12-bit depth resolution (0–4095), the exposure time was set such that the maximum value of $I_{w}\left(i,j\right)$ was approximately 90% of the dynamic range (4095) at each wavelength.

In NIR spectral measurements, variance such as non-specific scatter occurs on the surface of the sample. In this regard, the variance is reduced using the standard normal variate (SNV) for baseline correction of the spectrum as follows:

(3) $$Z\left(x\right)=\frac{x-\mathrm{mean}\left(x\right)}{\mathrm{std}\left(x\right)}$$

where ***x*** is a row vector containing the original spectrum, mean(***x***) is the mean of ***x***, std(***x***) is the standard deviation of ***x***, and Z(x) denotes the SNV-transformed spectrum. In this study, A(i,j) was assigned to ***x***.

### 2.5. Classification Algorithm

In this study, an artificial neural network with a dense multilayer was employed to identify tumors from OTN-NIR MSI data [25,26]. Each unit in a particular layer was connected to all the units in the next layer. The input value $z_{j}$ of the j th unit was calculated by adding the bias $b_{j}$ to the sum from 1 to the i th of the values, which is the multiplication of the output values $x_{i}$ and the corresponding weight $w_{ij}$ as follows:

(4) $$z_{j}=\overset{m}{\underset{i=1}{\sum }}w_{ij}x_{i}+b_{j}$$

where $z_{j}$ represents the input to the activation function, and the response is the output of the unit. As the activation function, the sigmoid and Rectified Linear Unit (ReLU) functions were used as follows:

(5) $$\sigma \left(z_{j}\right)\;=\;\frac{1}{1+e^{-z_{j}}}$$

(6) $$R\left(z_{j}\right)=\;\max\left(0,z_{j}\right)$$

As updating weights, the backpropagation method was used to train the neural network as follows:

(7) $$∆w_{i,j}^{k-1,k}=-\eta \frac{\partial E}{\partial w_{i,j}^{k-1,k}}\;$$

where $w_{i,j}^{k-1,k}$ is the weight between the i th unit in the (k−1) th layer and the j th unit in the k th layer, E is the error function, and η is the learning rate.

**Figure 5:**
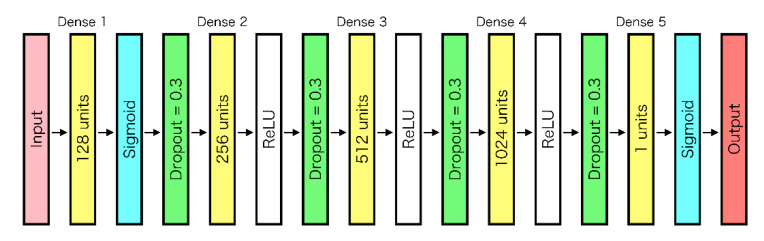
Neural network structure.

To suppress overlearning, a dropout process was applied to all layers except for the final layer. Adam was used as the optimization function. The structure of the neural network used in this study is shown in Figure 5. Because the final output value indicates the probability of discrimination as “tumor” from the multispectral input data of a pixel, the prediction results were displayed as binary classification with 60% of the cutoff value and heat map.

In this study, multispectral imaging data were extracted from the region of the back to the waist in mice before tumor implantation as “normal” teaching data. Multispectral imaging data of the tumor area were defined by medical experts and were extracted from mice 14 days after tumor implantation as “tumor” teaching data. To classify “normal” and “tumor”, leave-one-out cross-validation was employed. In this procedure, the pixels of a mouse were classified by data sets of eight mice built as teaching data. This procedure could provide an unbiased estimate of generalization ability.

The prediction accuracy was evaluated by classifying the pixels into four groups, namely: tumor predicted as tumor (true-positive: TP), tumor predicted as normal (false-negative: FN), normal predicted as tumor (false-positive: FP), and normal predicted as normal (true-negative: TN). From the classified pixels, the specificity, sensitivity, and accuracy can be calculated as follows:

(8) $$\mathrm{Specificity}\;\left(\%\right)=\frac{\mathrm{TN}}{\mathrm{FP}+\mathrm{TN}}\times 100,$$

(9) $$\mathrm{Sensitivity}\;\left(\%\right)=\frac{\mathrm{TP}}{\mathrm{TP}+\mathrm{FN}}\times 100,$$

(10) $$\mathrm{Accuracy}\;\left(\%\right)=\frac{\mathrm{TP}+\mathrm{TN}}{\mathrm{TP}+\mathrm{TN}+\mathrm{FP}+\mathrm{FN}}\times 100.$$

For binary classification of pixels in tumor-implanted mice, it was challenging to draw a correct boundary between the tumor and the normal areas. Therefore, the pixels that could be defined as tumor or normal were extracted, and the binary classification’s image, specificity, sensitivity, and accuracy were derived.

## 3. Results and Discussion

**Figure 6:**
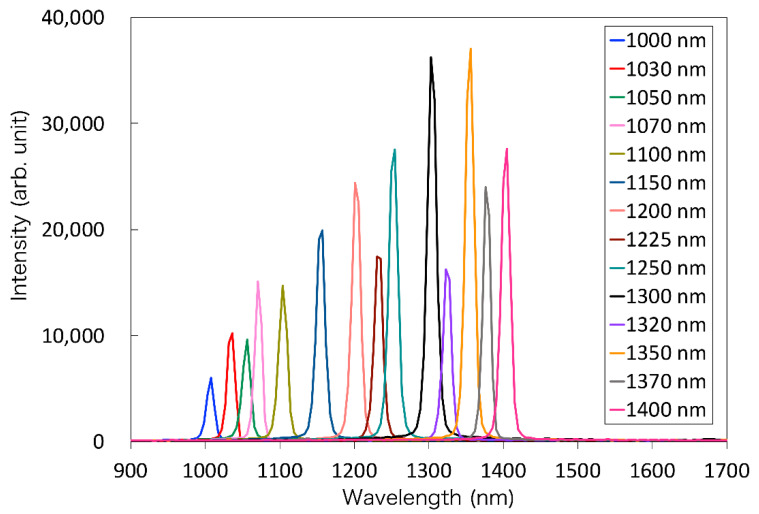
The spectrum of the transmitted light through each bandpass filter.

First, the NIR light transmitted through each of the 14 bandpass filters was detected by spectroscopy to validate the specifications of the filters. The results of the spectroscopy of the reflected light from the standard reflector are shown in Figure 6. Each bandpass filter was confirmed to perform as in the specifications. The system with these filters is available as the light source for MSI in the OTN-NIR wavelength range.

**Figure 7:**
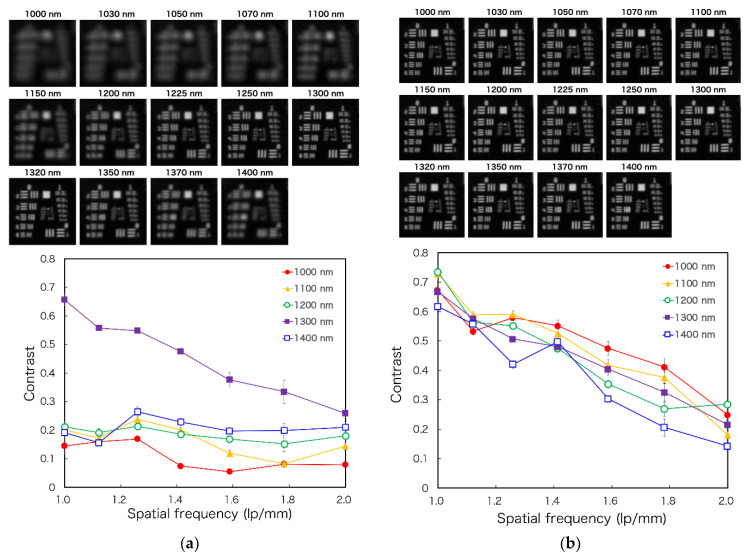
The figure displays 1951 USAF resolution chart images for the laparoscope at each spectral band (Top) and the spatial resolving power of the laparoscopic imaging system by a contrast transfer function analysis (Bottom): (**a**) without the tunable lens and (**b**) with the tunable lens.

The 1951 USAF resolution chart images for each wavelength were captured to evaluate the spatial resolution of the developed laparoscopic NIR imaging device, with or without a tunable lens. From these images, the contrast of each wavelength can be calculated. These images and the calculated contrasts are shown in Figure 7a,b. According to the Rayleigh criterion, line pairs can be considered resolved if the contrast value is higher than 0.26 [27]. Although the images are focused on around 1300 nm without the tunable lens, the tunable lens setup could focus on 14 wavelengths. Moreover, the results show that the image can be resolved up to 1.6–2.0 lp/mm at 1000–1400 nm wavelengths using the tunable lens. In future, custom-made achromatic lenses may be used to address the wavelength irregularities; however, this may present disadvantages for the laparoscopic application of MSI because the number of lenses and weight will increase, and the light intensity will decrease. In contrast, although the angle of view of the tunable lens needs to be corrected for each wavelength, it has the potential to be used for laparoscopic short-wavelength IR (SWIR) MSI, which has a broader range of wavelengths because it can correct the focus shift with a single lens.

**Figure 8:**
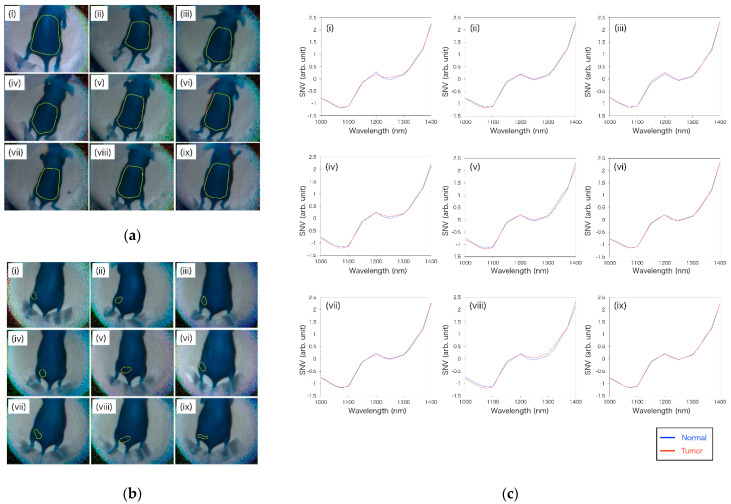
(**a**) Boundaries of pixels used as teaching data for the normal area before tumor implantation in nine mice; (**b**) boundaries of pixels used as teaching data for the tumor area of the nine tumor-implanted mice; (**c**) average of the standard normal variate (SNV)-processed spectrum of normal and tumor pixels in each mouse.

Through the 14-wavelength filter, the mice before and 14 days after implantation of the tumor could be captured by OTN-NIR MSI with live imaging. In this imaging, the average exposure time of each wavelength was 46 ms. Figure 8a,b shows the OTN-NIR pseudo-color images (B: 1000 nm, G: 1200 nm, R: 1400 nm) of each mouse with the boundaries of the pixels used in the normal and tumor datasets. In Figure 8c, the average SNV-processed spectra of normal or tumor datasets are shown. In this study, no pixels with $I_{w}\left(i,j\right)-I_{d}\left(i,j\right)$ lower than zero in the region of interest (ROI) were present. Although all the absorption spectra in Figure 8c show a similar shape, they also show differences between the normal and the tumor pixels at approximately 1070, 1250, and 1400 nm. This indicates that the OTN-NIR absorption spectra likely include tumor-specific information.

**Figure 9:**
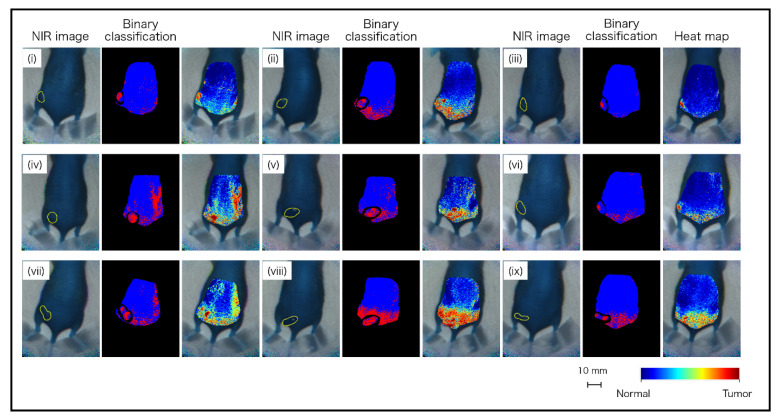
Classification images of tumor-implanted mice by neural network using OTN-NIR MSI datasets.

Based on these datasets, machine learning was performed using neural networks. The discrimination results of the nine mice are shown in Figure 9. It was visually apparent that the pixels identified as “tumor” and “normal” were aggregated in the tumor and normal areas, respectively. However, there were also some FP and FN pixels. In particular, various FP pixels were observed between the waist and tail. It is considered that the spectrum was similar between the tumor and waist, as the tumor was in the waist region. As shown in the heatmap diagram in Figure 9, although there were red level pixels that were false-positive, there was a tendency for a large amount of red level pixels to be in the tumor area and a small number of red level pixels in the normal area. In the analyzed results, total pixels (102,420 px) were classified as follows: TP, 2629 px; FN, 2287 px; FP, 10,212 px; and TN, 87,292 px. From the classified pixels, the specificity, sensitivity, and accuracy were calculated as 89.5%, 53.5%, and 87.8%, respectively. The results are listed in Table 2.

In this study, although aggregated TP pixels were visually recognized and confirmed in all tumor-implanted mice, identification of tumor-implanted mice showed lower sensitivity than hyperspectral imaging in previous studies [28]. The reasons for these results are considered to be the following: (i) As the imaging time exceeded the order of seconds, there were blurred pixels in the merged multidimensional image due to breathing and heartbeat. (ii) The data set size was lower than that of the previous study because of the smaller number of mice and small ROI. (iii) Because the illumination was non-uniform compared with that of the conventional method, more pixels of halation and dark shadows were included. (iv) Although the previous study used an NIR wavelength of 256 bands, the most specific absorption wavelengths for tumor discrimination were different because only 14 wavelengths were used in this study.

As for the above, future prospects are considered as follows: (i) The time required for imaging was long because the filter change was done manually. By automating the filter change, the total imaging time could be shorter than that in this study. (ii) The proposed improvements noted above enable image capture at high speeds; thus, several sample images with various magnifications could be acquired. (iii) A suitable FOV for identification could be created by imaging from various angles, and halation and dark shadow could be decreased by changing the arrangement of the illumination at the tip of the laparoscope. (iv) The valuable wavelengths for the identification of tumors could be selected from NIR-HSI data using resected specimens. In this study, OTN-NIR MSI was investigated using commercially available filters via straightforward construction; however, spectral images with high wavelength resolution could be obtained by adopting an acoustic tunable filter (AOTF) and a liquid crystal tunable filter to select the wavelength before imaging or light output [21,29,30]. In particular, a combination of an AOTF and a supercontinuum light source is promising because high power output and fast wavelength switching can be obtained. (v) In addition to the points mentioned, although a neural network was used as a machine learning method in this study, other methods such as support vector machine and principal correlation analysis have been proposed to identify lesions [11]. The identification accuracy may be improved by investigating the optimal algorithm.

Although the subcutaneous tumor model of mice was adopted because it was straightforward to handle the tumor implantation, the overall sensitivity was low, and two out of nine samples showed a sensitivity below 30%. The conceivable reasons are that light scattering by the skin, which has a complex structure such as hair follicles, resulted in the inclusion of the spectra disruption through the skin in all tumor data. In addition, because it was difficult to make the conditions uniform for the specimens (e.g., tumor size and contrast of subject), it may be observed that the identification ability was low in some samples that included individual differences. In contrast, gastrointestinal tissue composed of smooth muscle may be a suitable target for spectral imaging because it has a simpler structure than skin and shows less optical scattering [31]. Therefore, the proposed device is expected to be applied to the gastroenterological field. In particular, there is a demand in medical applications to visualize the location of invisible digestive tract cancer from the serous membrane side during laparoscopic surgery. Previous studies have indicated that tumors existing under normal tissue in the stomach could be distinguished by high sensitivity, specificity, and accuracy at four wavelengths [17,19]. Thus, the analysis of resected digestive tract cancer specimens by NIR-HSI on the serous side is underway. By determining the effective wavelengths for cancer detection, processing techniques such as those used for tissue oxygen saturation may be applied [32,33]. Therefore, laparoscopic OTN-NIR MSI has the potential to enable cancer detection in deep regions during surgery.

## 4. Conclusions

In this study, we developed a laparoscopic imaging device and a wavelength-selectable light source to perform OTN-NIR MSI, which was performed on nine live mice before and after tumor implantation. The images of tumor-implanted mice were classified using normal and tumor pixel datasets using a neural network. As a result, pixels of subcutaneous tumors were distinguished without opening the skin. The specificity, sensitivity, and accuracy were 89.5%, 53.5%, and 87.8%, respectively. This is the first report demonstrating the classification of living tissues with OTN-NIR MSI under laparoscopy. Therefore, this study serves as a preliminary step in clinical research on laparoscopic OTN-NIR MSI for future use by optimizing the algorithm and imaging conditions.

In future, the use of a high-resolution NIR camera and light source that can select wavelengths automatically will enable the acquisition of a large amount of OTN-NIR MSI data at high speed. Therefore, the device may realize higher discrimination accuracy. In addition, the device is expected to be used in various situations, such as laparoscopic surgery.

## Figures and Tables

**Table 1:** Bandpass filter information and the tunable lens settings.

Wavelength (nm)	Optical Power (dpt)	FWHM (nm)	Vendor	Model Number
1000	−2.03	10	Edmund Optics	65-782
1030	−1.87	10	Thorlabs, Inc	FLH1030-10
1050	−1.55	10	Edmund Optics	65-783
1070	−1.61	10	Thorlabs, Inc	FBH1070-10
1100	−1.42	10	Edmund Optics	65-784
1150	−0.85	10	Edmund Optics	65-785
1200	−0.69	10	Edmund Optics	65-786
1225	−0.29	10	IR System Co., Ltd.	NB-1225-010 nm
1250	−0.22	10	Edmund Optics	65-787
1300	0.29	12	Edmund Optics	65-788
1320	0.59	12	Thorlabs, Inc	FB1320-12
1350	0.67	12	Edmund Optics	65-789
1370	0.94	10	IR System Co., Ltd.	NB-1370-010 nm
1400	1.18	12	Edmund Optics	65-790

**Table 2:** Prediction results of OTN-NIR multispectral imaging (MSI) analysis for the tumor-implanted mice.

No.	Tumor Volume (mm^3^)	Tumor (px)	Normal (px)	Specificity (%)	Sensitivity (%)	Accuracy (%)
i	174.0	360	11,015	96.3	54.7	95.0
ii	526.0	507	11,527	89.4	53.5	87.9
iii	355.2	451	10,923	99.2	26.6	96.3
iv	416.1	565	10,322	80.9	72.9	80.5
v	821.9	735	8698	88.0	61.1	85.9
vi	276.5	558	12,472	94.6	21.3	91.5
vii	696.2	648	9453	87.1	52.3	84.9
viii	419.5	684	10,457	77.8	74.1	77.6
ix	341.9	408	12,637	90.0	52.7	88.8
total	-	4916	97,504	89.5	53.5	87.8

## Data Availability

The data presented in this study are available on request from the corresponding author.
